# The Association between Demographic Characteristics, Lifestyle Health Behaviours, and Quality of Life among Adolescents in Asia Pacific Region

**DOI:** 10.3390/ijerph16132324

**Published:** 2019-07-01

**Authors:** Regina L.T. Lee, Wai Tong Chien, Keiko Tanida, Sachi Takeuchi, Phuphaibul Rutja, Stephen W. H. Kwok, Paul H. Lee

**Affiliations:** 1 Faculty of Health and Medicine, School of Nursing and Midwifery, The University of Newcastle, Callaghan 2308, New South Wales, Australia; 2 The Nethersole School of Nursing, Faculty of Medicine, The Chinese University of Hong Kong, Hong Kong, China; 3 College of Nursing Art and Science, University of Hyogo, World Health Organization Collaborating Center for Nursing in Disasters and Health Emergency, Hyogo 13-71 Kitaoji-cho, Japan; 4 Ramathibodi School of Nursing, Faculty of Medicine, World Health Organization Collaborating Center, Krung Thep Maha Nakhon 10400, Thailand; 5 School of Nursing, World Health Organization Collaborating Center for Community Health Services, The Hong Kong Polytechnic University, Hong Kong, China

**Keywords:** adolescents, lifestyle health behaviours, quality of life, demographic characteristics, Asia Pacific region

## Abstract

*Background*: Given the risk of physical and psychosocial health that emerge in adolescents that are continuing into adulthood, identifying and addressing early signs of health-related quality of life (HRQoL) decline provides an opportunity to ensure that young people have a healthier progression through adolescence. *Aim*: To investigate the association between demographic characteristics, lifestyle health behaviours, and HRQoL of adolescents who live in Asia Pacific region, including Hong Kong in China, Beijing in China, Akashi in Japan, Seoul in South Korea, and Bangkok in Thailand. *Methods*: A cross-sectional self-reported survey carried out in a sample of 2296 adolescents that were aged 9–16 years (mean= 12.0; standard deviation [SD] = 1.63) was conducted in the five cities of the Asia Pacific region between January and August 2017. Demographic characteristics, adolescent lifestyle behaviours, and HRQoL were measured with demographic questionnaire, Adolescent Lifestyle Questionnaire’s (ALQ) seven domains and Paediatric Quality of Life’s (PedsQL 4.0) global score, respectively. Mixed multilevel model (MMLM) was used to fit the data. *Results*: After adjusting the demographic variables, one score increase in ALQ physical participation, nutrition, social support, and identity awareness are associated with an increase in PedsQL global score. On the contrary, one score increase in ALQ health practices is associated with a decrease in the PedsQL global score. The estimated mean of PedsQL global score of South Korea, Beijing, and Japan were better, while the score of Thailand and Hong Kong were poorer. *Conclusions*: Differentiating the impacts of promoting health behaviours among different countries can help in better understanding the health needs of adolescents in each country, especially in the Asia Pacific region, so that adequate and relevant resources can be allocated to reduce health-risk taking behaviours among this vulnerable group for health-promoting strategies.

## 1. Introduction

Healthy People 2020 used self-reported outcome measurements to assess the positive aspects of a person’s life, such as life satisfaction and emotional wellbeing [1]. The focus in the measurement of health, especially for non-communicable diseases (NCDs), has been much broadened for the past two decades in the public health agenda. The global prevention and control of NCDs was targeted at the Sustainable Development Goal (SDG) 3 [2]. There is an increasing number of studies aimed at recognizing the health-related quality of life (HRQoL) as an important health outcome measurement related to NCDs, such as health behaviours in the adolescent population [3,4], especially in the needs assessment for service delivery and in an evaluation of the impact of public health agenda of this vulnerable group [5]. HRQoL is a multi-dimensional concept that focuses on the impact health status on individual wellbeing and Paediatric Quality of Life 4.0 (PedsQL 4.0) has four aspects of functioning, including physical, mental, emotional, and social [6].

Adolescence is a unique developmental group in the transition from both childhood and adulthood exposed to risk-taking behaviours and barriers to accessing health services [7]. Recognizing HRQoL associated factors can generate relevant information for public health policy to promote the health and wellbeing of adolescents.

A total of 36 million death (63%) were due to non-communicable diseases such as cardiovascular diseases, diabetes, chronic respiratory diseases and cancer in South-East Asia and other Western countries and they were strongly associated with physical inactivity, unhealthy diet, and substance use [8]. According to World Health Organization (WHO), adolescent health is one of the main focuses to improve global health, since poor or undesirable health and related behaviours in adolescents can contribute to an adverse or negative health condition in their adult life [9,10].

In this fast-changing world, globalization and advanced technology have challenged the adolescents’ lives continuously by bombarding them with high expectations and social norms from the world culture. During puberty, adolescents strive to search for their own identity and to cope in this multicultural world. There was evidence reporting that adolescents’ health could be affected by the socio-economic factors, personal characteristics and demographics such as education, family income, social support, economic status, and health policies [11,12]. It is important to identify the associations between these demographic factors, health behaviours and quality of life (QL) in order to plan appropriate programs and services for this vulnerable group [13].

The multi-dimensional aspects affect adolescents’ life, including health status, lifestyle behaviours, emotional wellbeing, interpersonal relationship, and socioeconomic factors, such as family support and risk-taking behaviours [14]. During the past decade, some of the studies examined the predictors or determinants of healthy lifestyle behaviours and psychosocial wellbeing among adolescents [15,16]. Some identified factors were classified into four categories: (1) lifestyle health behaviours (e.g., physical participation and sedentary life), (2) psychosocial health (e.g., self-efficacy, anxiety, and depression), (3) socio-economic and socio-demographic factors, and (4) demographic variables (e.g., education and household income) [17]. Lifestyle behaviours and socio-demographic variables of adolescents can also predict or mediate their perceived QL. Studies found that obese adolescents that are associated with their poor lifestyle behaviour and/or psychosocial health condition might result in lower self-reported QL when compared to healthy adolescents [18,19]. Psychosocial health condition is associated with QL among adolescents. Depression was found as a dynamic and co-evolving factor in early years of adolescence and it could be strongly related to later substance use [20].

Concerning the physical health status and mental well beings of adolescents in relation to their QL in the Asia Pacific region, a cross-cultural study reported that adolescents across four countries (Hungary, Poland, Turkey and the United States) with strong optimistic self-belief and self-efficacy performed physical activities more frequently [21]. However, health status and physical activities are highly evidenced to be the determinants in adolescents’ and adults’ perceived QL. Another cross-cultural study on adolescents’ QL reported that physically active adolescents have higher self-reported QL total scores; whereas, boys were more physically active [22]. Lastly, socio-economic status can also affect QL among adolescents. A study found that adolescents with higher socio-economic status had better health status, and in turn, lead to an enhanced QL [23].

While most of the studies have focused on vulnerable social groups with specific diseases, such as sexually transmitted diseases; chronic physical and mental health problems; or, risk-taking behaviours, such as substance use and childhood obesity, there are limited studies with relatively large-scale population-based design examining comprehensively on personal, physical, and psychosocial factors that influence adolescents’ quality of life in Asian communities. In addition, the Healthy People 2020 consists of 467 objectives, in which one-fourth pertains to adolescent health [24]. Two main goals have also been set: “Promoting quality of life, healthy development and healthy behaviour across life stages” and “Creating social and physical environments that promote good health” [25].

Behavioural factors and personal life have been considered as the predictors for the individual’s quality of life in the adolescent population, as they are at a critical time in their life to make independent health care decisions. According to the Pender’s Health Promotion Model (HPM) [26], individuals have unique variables that affect their actions. Pender’s HPM identified some of the predisposing factors, such as perceived benefits and barriers to predict health behaviours. In the HPM, the psychological factors include personal strengths and difficulties, depression, anxiety, and stress, and the social factors include peer and family support are used to predict health behaviours, perceived social support, and behavioural factors, such as adolescent lifestyle, which may affect QL. Self-caring in diabetes includes personal, psychological, and social factors, with the associated cognition and perception, help health services suppliers to plan and carry out desirable intervention to promote diabetic self-management behaviours [27]. Perceived benefits and barriers have an important role in the self-care process among diabetes based on HPM. Glasgow, Toobert, and Gillette found that there was a significant, but reverse, relationship between perceived barriers and self-caring behaviours [28]. Psychological barriers are important factors in self-management behaviours. The relationships between the proposed predictors and health behaviours, especially for adolescents based on HPM, remain unclear.

Adolescents with different ethnic and cultural backgrounds may have different health promoting behaviours, which are associated with QL, as discussed and supported by studies that focused on cross-sectional and cross-cultural surveys that specifically targeting adolescents. Limited studies have examined the relationship between lifestyle behaviours and the quality of life among adolescents from different ethnic groups in the Asia Pacific region.

Differentiating the impacts of health-promoting behaviours among different countries can help in better understanding the health needs of adolescents in each country, especially in the Asia Pacific region, so that adequate and relevant resources can be allocated to this vulnerable group for health promoting strategies.

The aim of this study was to investigate the associations between demographic characteristics, seven domains of adolescent lifestyle questionnaire (ALQ), and the total Global Score of the PedsQL 4.0 of adolescents who lived in the five cities of the Asia Pacific region, including Hong Kong in China, Beijing in China, Akashi in Japan, Seoul in South Korea, and Bangkok in Thailand.

## 2. Methods

### 2.1. Sampling

This is a multi-site cross-cultural and cross-sectional quantitative study design. It is a self-reported survey of adolescents in five cities of Asia Pacific region including Hong Kong in China, Beijing in China, Tokyo and Hyogo in Japan, Seoul in South Korea, and Bangkok in Thailand between January and August 2017. The multi-site researcher team members had held more than ten teleconference meetings to finalize the resulting protocol. The inclusion criteria of the study samples were students studying in grade 4 to 9 in primary and secondary schools. Students were conveniently recruited in each city. Each city recruited their sample based on the study inclusion and exclusion criteria.

### 2.2. Sample Size Calculation

The sample size that was required in each city was calculated based on the population size aged 10–14 ($n_{10–14}$), 15–19 ($n_{15–19}$), and all age ($N_{country}$) in each country (region) [29,30,31,32,33], as well as population size of all age ($N_{city}$) in each city [29,34,35,36,37]. The population size aged 10–16 is $n_{10–16}=\left(n_{10–14}+n_{15–19}\times 0.4\right)/1.4\times \left(N_{city}/N_{country}\right)$. The sample size is $n=\Omega ∕\left(1+\Omega ∕n_{10–16}\right)$ [38], where $\Omega =z^{2}\times p\left(1-p\right)÷e^{2}$. The *z* = z-score, set at 1.96 where confidence level at 0.95; *p* = response distribution, set at 0.5; *e* = margin of error, set at 0.05. Thus, the target sample sizes of Hong Kong, Beijing, Akashi, Seoul, and Bangkok were 384, 385, 374, 384, and 384, respectively.

### 2.3. Study Tools

The self-reported survey included a socio-demographic date sheet, Pediatric Quality of Life Inventory 4.0 (PedsQL 4.0) [4], and Adolescent Lifestyle Questionnaire (ALQ) [39]. They were translated into each country’s language (Chinese, Japanese, Korean, and Thai) prior to administering to the target sample. The translations of study instruments followed a standardized translation protocol [40] and they have been proven to be valid and reliable in many countries, including in Chinese, Thai, Japanese, and South Korean samples [41,42,43,44].

The participants’ socio-demographic characteristics measured included gender, age, grade, religious belief, frequency of joining religious service, father’s education level, mother’s education level, and parents’ marital status.

Quality of life was measured by the Pediatric Quality of Life Inventory 4.0 (PedsQL 4.0) Generic Core Scales that focus on physical, emotional, social, and school functions, which is a modular instrument for measuring health-related quality of life (HRQL) in children and adolescents ages 2–18 [6]. It is applicable for healthy school and community populations. The PedsQL 4.0 consists of 23 items that were validated in the multicultural specific survey in Asia Pacific region [6]. The instrument contained four domains, namely (1) physical functioning (eight items), (2) emotional functioning (five items), (3) social functioning (five items), and (4) school functioning (five items). It is a self-report questionnaire. Each negative keyed item was measured with a five-point Likert scale that ranged from “1=never” to “5=almost always”. The domain scores were the sum of all ordinal scales in a domain. The total PedsQL 4.0 global score was the average of all the domain scores. The raw scores were reversed in this study, so that higher score indicated a better quality of life and its Cronbach’s alpha was 0.804, which was satisfactory [6]. The PedsQL 4.0 had already been translated into multiple languages [28]. It had demonstrated a fair to good reliability and validity in different age-appropriate populations in various culture, including Chinese [41], Korean [42], Japanese [43], and Thailand [44].

The lifestyle health behaviours in this study were measured with Adolescent Lifestyle Questionnaire (ALQ) [39]. The ALQ consisted of seven domains and 43 positive keyed items, and it was a Likert-type self-reported survey with seven domains: identity awareness, nutrition, physical participation, safety, health awareness, social support, and stress management. Each positive keyed item was measured with a five-point Likert scale that ranged from “1=never” to “5=almost always”. The total score represented the sum of the dimensions. The global ALQ score was the average score of all ordinal scales. A higher score indicated better lifestyle behaviours. The ALQ had a Cronbach’s alpha of 0.93 for the total ALQ; the dimension coefficients ranged from 0.60–0.87. The Cronbach’s alpha for the Chinese version of the ALQ was 0.92, and the alpha coefficients for the seven dimensions ranged from 0.60–0.83.

### 2.4. Ethical Approval

Ethical approval was obtained from their institution in each city. Ethics approvals were obtained from either the Institute’s Research Ethics Committee or Institute Review Board (School Research Committee in Hong Kong and Beijing, Institutional Review Board in Thailand and South Korea, Ethic Committee, the University of Hyogo in Japan) prior to conducting the data collection. Written parental informed consents were obtained through each school’s communication system with the parents in each city. There was a total of 2296 written parental informed consents obtained. The purpose of study and the tools were verbally explained to the students, their parents, and the school principals with an information sheet. Privacy and confidentiality were ensured; personal information was not disclosed, and was handled by the researchers of this study.

### 2.5. Data Collection

The research team in each city provided a briefing to the school teachers and parents via the study information sheet with the aims of the study. The students could withdraw at any time without penalty. School teachers distributed the questionnaires after obtaining parental consent. They arranged the logistics of distributing and collecting surveys to the target population in each city. The students were encouraged to complete the surveys at school and to answer demographic questions, such as family income at home. The school teachers collected the return questionnaires from students and passed to the research team member. They also addressed any questions and provided support to the school teachers and parents when necessary.

### 2.6. Statistical Analysis

IBM SPSS Statistics for Windows, Version 25.0. (Armonk, NY: IBM Corp.) was used for data analysis. Mixed multilevel model (MMLM) was used to fit the data. The dependent variable was the PedsQL 4.0 Global Score. Normal distribution was assumed for target variables and the identity link function was specified.

With regard to the fixed effects, demographic variables with small missing percentages were included in the model-only variables with missing percentage <10% were analyzed. City, age, gender, education level, religious belief, and parents’ marital status have percentages of missing data <10%, and therefore included. Moreover, adolescent lifestyle is considered as an independent variable. Therefore, ALQ physical participation, ALQ nutrition, ALQ social support, ALQ stress management, ALQ identity awareness, ALQ health practices, and ALQ safety were specified as fixed effects.

The intercepts of cities were specified as a random effect. The covariance type of random intercept was specified as diagonal. A robust covariance estimator was used.

The residual method was used to calculate *df*. Pearson residuals were checked in boxplots between cities, in Q-Q plot, and in scatterplot against predicted values and locally estimated scatterplot smoothing (LOESS) curve calculated with Epanechnikov’s kernel. There were no significant violations of the assumptions of independence of observations, normality, homoscedasticity, and linearity. Cases with an absolute value of Pearson’s residual > 4 were excluded in the first model and the second model was rerun.

## 3. Results

### Demographics

The respondents resided in Hong Kong (*n* = 367), Beijing (*n* = 395), Tokyo and Hyogo (*n* = 869), Seoul (*n* = 310) and Bangkok (*n* = 355). Their age ranged between 9 and 16, and the mean is 12 (*SE* = 0.034). There were 52% male. They studied in primary 4 to secondary 3. Over 65% of them did not have a religious belief. Over 26% of them were protestants. Nearly 85% of the parents were married and living together.

Table 1 shows the results of the F test on the fixed effects of demographic characteristics and ALQ domains. The fixed effects of City, Education level, religion, ALQ physical participation, ALQ nutrition, ALQ social support, ALQ identity awareness and ALQ health practices were significant.

Table 2 shows that after adjusting the demographic variables, one score increase in ALQ physical participation, ALQ nutrition, ALQ social support and ALQ identity awareness are associated with an increase in PedsQL 4.0 global score. On the contrary, one score increase in ALQ health practices is associated with a decrease in PedsQL 4.0 global score.

**Figure 1:**
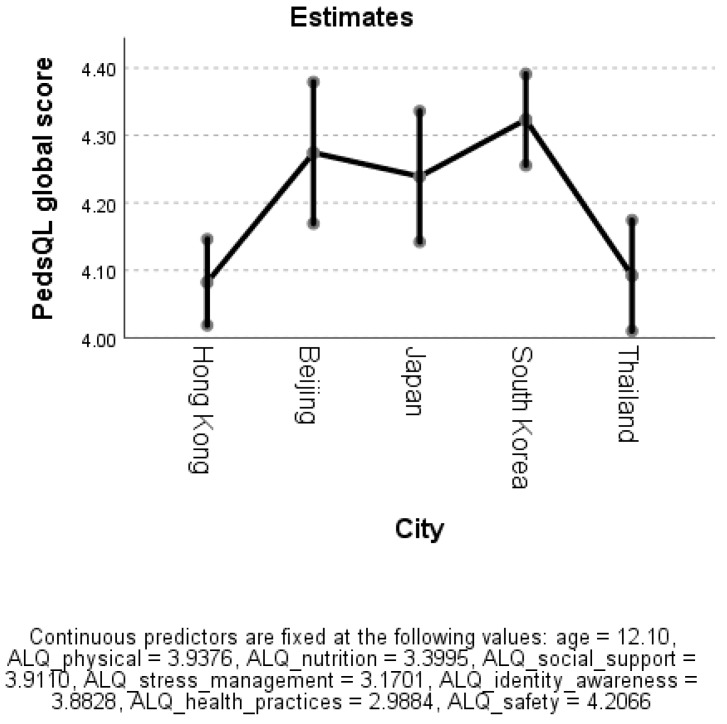
Estimated means of PedsQL 4.0 global score.

Table 3 and Figure 1 shows that the estimated mean of PedsQL 4.0 global score of South Korea, Beijing and Japan ranged between 4.2 and 4.3, while the score of Thailand and Hong Kong were 4.1 respectively.

## 4. Discussion

This cross-sectional self-reported survey aimed to investigate the associations between demographic characteristics, seven domains of the ALQ and the Global Score of the PedsQL 4.0, while controlling for geographical locations, gender, education level and parents’ marital status in the five cities of the Asia Pacific region.

The findings indicate the association between individual sociodemographic characteristics and QL. The results of this study are similar to the study conducted by the World Health Organization (WHO) with 43 countries to evaluate the adolescents’ lifestyles at the national level in order to identify the cultural variations of the lifestyle patterns among the adolescents and provide support for the country to deliver appropriate services and programs to monitor the habits of this target population and plan health prevention and promotion policies [45].

In this study, the four domains of the ALQ included physical participation, nutrition, social support and identity awareness and were positively associated with the PedsQL 4.0 global score. The ALQ’s health practices domain was negatively associated with the PedsQL 4.0 global score while controlling the city, gender, education level and parents’ marital status.

General health practice domain was negatively associated with quality of life. Items of ALQ’s health practice domain included “Report unusual body changes (item 18)”, “Talk to teacher or nurse re ways to improve my health (item 31)”, “Read magazines about health topics (item 32)”, and “Discuss health issues with others (item 33)”. A higher frequency of such behaviours may imply poorer physical health status which is associated with lower QL. The result of this study is consistent with the findings reported for adolescent health projects in the south of Portugal and Spain that there were cross-cultural differences in the perception of adolescents’ quality of life [46].

ALQ’s stress management and safety behaviours domains were not significantly associated with the PedsQL 4.0 global score. Items of ALQ’s stress management domain included “Exercise to control my stress (item 26)”, “Talk to my friends about my stress (item 27)”, “Use helpful strategies to deal with stress (item 37)”, and “Use spiritual beliefs to deal with stress (item 39)”. Adolescents who have a higher frequency of experiencing stress may practice stress management more frequently to maintain QL.

Items of ALQ’s safety behaviours domain included “Wear seatbelts in automobile (item 4)”, “Avoid doing drugs (item 7)”, “Refuse a drive if the driver is drinking (item 11)”, “Avoid tobacco products (item 17)”, and “Avoid alcohol (item 30)”. Adolescents who practice safety behaviours may be disciplined or disciplining themselves for examples avoiding tobacco, alcohol and drug abuse. Its association with QL may need further study.

The results of this study show the association between the demographic characteristics and adolescents’ lifestyle behaviours which were the contributing factors to QL. This finding is similar to the study reported among adolescents’ lifestyle behaviours, that the socio-demographical factors were the predisposing factors reported in the Hong Kong and Guangzhou studies [13]. Although the health issues of adolescents in the region and worldwide may not be similar, it is important to identify the predisposing and contributing factors to those unhealthy and risk-taking behaviours in each country in order to provide cultural specific early intervention and appropriate management in this sensitive changing world which impact the quality of life of adolescents in the countries of the Asia Pacific region [47]. The influence of technology on globalization and adolescents’ cultural and global identity is critical as adolescents faced many different cultures in their daily lives. Future intervention must be culturally specific and tailored.

Studies have reported the association between mental health status and QL among adolescents [48,49]. In contrast, this study identified predictors such as lifestyle behavioural factors and personal life characteristics among the adolescents in the school communities that live in the five cities of the Asia Pacific region. The findings of this cross-sectional self-reported survey on adolescent health provided an overview of the relationships between the social determinants in quality of life programs and service development on promoting the psychosocial health conditions of adolescents in the region by identifying those contributing factors to determine adolescents’ QL in specific culture.

More specifically the NCD strategy aims to reduce behavioral risk factors; improve health literacy; develop a health promoting environment; improve equity in access to health promotion and prevention; reduce the proportion of the population at increased risk of disease; improve the quality of life and reduce the need for care”.

The results of the study may contribute to a national portrait on QL for the youth developmental framework in the Asia Pacific region. This is similar to the WHO’s development of Adolescent Health Global Framework as a guide for planning adolescent health programs and services related to health prevention and promotion activities in 2017 [45]. The study findings can provide new evidence on the strategic plan improving the quality of paediatric care by identifying factors that could be modified using action research and evaluation specifically designed to optimize program efficiency and impact [50]. Implications specifically to the Asia Pacific region would be based on the findings of this regional adolescent health project for health providers to promote positive outcomes for healthy youth development and identify the current available cultural-specific health-related quality of life instruments [51]. The cross-cultural study findings are similar to the commission on adolescent health and wellbeing that aimed to promote positive youth development holistically in order to enhance their competence during development for a better quality of life at both the country and global levels [52]. Thus, the findings of this study have added new evidence on the early identification of risk factors affecting adolescents’ QL that is critical in addressing the psychosocial health conditions of adolescents especially during the developmental stages and providing relevant early interventions to this target group in specific cultural groups. In Australia, the longstanding challenge for the governments to improve the health status of indigenous group of Australian that has been identified as a human rights concern by United Nation committees [53].

Nonetheless, the study had its limitations. First, random sampling was not adopted in this study. There may be selection bias inherited in convenience sampling method. Some variables were not being measured in certain cities. There may be items which were not being asked in certain cities. It should be prevented if the survey should be repeated. This reflected points to be improved in the survey and a larger scale of the survey with good control of missing items is warranted. The missing of Family income, Father’s education level and Mother’s education level were >10%. It might indicate that adolescents were not familiar with their parents’ education level and family income. Therefore, one’s personal information including parents’ education level and family income should be reported by parents.

Differences in QL levels and predicting factors of QL among ethnic groups underscore the need to target population segments to promote wellbeing better or differentially. More work is needed to explore adolescents’ QL and the associations to their demographic characteristics and personal in relation to ethnicity.

## 5. Conclusions

This cross-sectional, cross-cultural and self-reported study confirmed that the personal characteristics and lifestyle health behaviours related to the predictors of adolescents’ QL in the Asia Pacific region. The study findings add new evidence to support the associations between personal characteristics, lifestyle health behaviours and QL among adolescents aged 9-16 years old, so that health planners, health educators and health promoters can plan and implement relevant programs and services to the specific cultural group to enhance their QL. Similar cross-sectional and cross-cultural study to examine the adolescent’s lifestyle behaviours and mental wellbeing among five cities have not been done previously. Thus, the findings of this regional adolescent health project should be disseminated both regionally and globally for the WHO Offices so that researchers plan more tailored culturally relevant health promotion interventions to enhance the QL for this population. However, the implications should be made specifically applicable to the WHO in the Western Pacific Region in assessing, planning and evaluating the health programs and health care services to enhance the QL for the adolescents that live in the Asia Pacific region.

## Figures and Tables

**Table 1:** *F* tests of fixed effects of demographic characteristics and adolescent lifestyle questionnaire (ALQ) domains.

Source	*F*	*df1*	*df2*	*p*
Corrected Model	224.022	4	1589	<0.001
City	224.022	4	1589	<0.001
Age	0.087	1	1589	0.768
Gender	0.621	1	1589	0.431
Education level	40.192	4	1589	<0.001
Religion	3.855	4	1589	0.004
Parents’ marital status	0.655	1	1589	0.418
ALQ physical participation	6.013	1	1589	0.014
ALQ nutrition	27.710	1	1589	<0.001
ALQ social support	6.327	1	1589	0.012
ALQ stress management	0.013	1	1589	0.910
ALQ identity awareness	501.718	1	1589	<0.001
ALQ health practices	9.635	1	1589	0.002
ALQ safety	0.010	1	1589	0.921

**Table 2:** Fixed Coefficients of fixed effects on Paediatric Quality of Life 4.0 (PedsQL 4.0) global score.

Model Term	Coefficient	*SE*	*t*	*p*	95% CI	
					Lower	Upper
Intercept	2.843	0.1584	17.943	0.000	2.532	3.154
Hong Kong	−0.010	0.0105	−0.937	0.349	−0.030	0.011
Beijing	0.182	0.0363	5.025	0.000	0.111	0.253
Tokyo & Hyogo	0.147	0.0334	4.404	0.000	0.082	0.212
Seoul	0.231	0.0135	17.182	0.000	0.205	0.258
Bangkok	0 ^b^	.	.	.	.	.
Age	−0.004	0.0143	−0.295	0.768	−0.032	0.024
Male	0.015	0.0196	0.788	0.431	−0.023	0.054
Female	0 ^b^	.	.	.	.	.
Grade 4	−0.124	0.0879	−1.410	0.159	−0.296	0.049
Grade 5	−0.092	0.0695	−1.327	0.185	−0.229	0.044
Grade 6	−0.094	0.0739	−1.275	0.202	−0.239	0.051
Grade 7	−0.151	0.0522	−2.896	0.004	−0.254	−0.049
Grade 8	−0.024	0.0786	−0.311	0.756	−0.179	0.130
Grade 9	0 ^b^	.	.	.	.	.
Protestantism	−0.064	0.0223	−2.863	0.004	−0.107	−0.020
Catholic Church	−0.051	0.1616	−0.313	0.754	−0.368	0.266
Buddhism	−0.098	0.0334	−2.939	0.003	−0.163	−0.033
Islam	−0.026	0.1071	−0.239	0.811	−0.236	0.184
Other religion	−0.246	0.0166	−14.785	0.000	−0.279	−0.213
No religious belief	0 ^b^	.	.	.	.	.
Parents not living together	0.027	0.0335	0.810	0.418	−0.039	0.093
Parents married and living together	0 ^b^	.	.	.	.	.
ALQ physical participation	0.049	0.0198	2.452	0.014	0.010	0.087
ALQ nutrition	0.073	0.0138	5.264	0.000	0.046	0.100
ALQ social support	0.112	0.0445	2.515	0.012	0.025	0.199
ALQ stress management	0.002	0.0200	0.114	0.910	−0.037	0.042
ALQ identity awareness	0.171	0.0077	22.399	0.000	0.156	0.186
ALQ health practices	−0.034	0.0110	−3.104	0.002	−0.056	−0.013
ALQ safety	−0.001	0.0123	−0.100	0.921	−0.025	0.023

^b^ This coefficient is set to zero because it is redundant.

**Table 3:** Estimated means of PedsQL 4.0 global score.

City	Mean	*SE*	95% Confidence Interval	
			Lower	Upper
Hong Kong	4.082	0.033	4.018	4.146
Beijing	4.274	0.054	4.169	4.379
Japan	4.239	0.049	4.142	4.336
South Korea	4.323	0.035	4.255	4.391
Thailand	4.092	0.042	4.010	4.174
